# Comparison of plasma and tissue levels of ZD1694 (Tomudex), a highly polyglutamatable quinazoline thymidylate synthase inhibitor, in preclinical models.

**DOI:** 10.1038/bjc.1998.37

**Published:** 1998

**Authors:** G. W. Aherne, E. Ward, N. Lawrence, D. Dobinson, S. J. Clarke, H. Musgrove, F. Sutcliffe, T. Stephens, A. L. Jackman

**Affiliations:** CRC Centre for Cancer Therapeutics, Institute of Cancer Research, Sutton, UK.

## Abstract

ZD1694 (Tomudex, raltitrexed) is a specific quinazoline antifolate thymidylate synthase inhibitor that relies on polyglutamation for high potency. Antibodies to ZD1694 have been used to establish a sensitive radioimmunoassay as an alternative to high-performance liquid chromatography (HPLC). The radioimmunoassay is reproducible, accurate and provides a means of determining low levels of ZD1694 in plasma (< 1 nM). By virtue of the high cross-reactivity of the antibodies with polyglutamated forms of ZD1694, it is also possible to measure the total concentration of drug in tissues. Results obtained in L1210 mouse leukaemia cells and in mouse tissues were similar to those previously determined using radiolabelled drug. Pharmacokinetic studies in mice have confirmed that the compound is rapidly eliminated from the plasma and that there is a prolonged terminal elimination phase. ZD1694 was measured in plasma (0.56 ng ml(-1); 1.2 pmol ml(-1)) up to 7 days after a single i.p. dose of 100 mg kg(-1) ZD1694. Liver, kidney and gut epithelium had a substantially higher level of ZD1694 immunoreactivity than plasma. For example, 24 h after a single i.p. dose at 1, 10 and 100 mg kg(-1), total drug levels in the liver were 480, 325 and 152 times higher than plasma levels respectively. In kidney and gut epithelium, total drug levels at these doses were approximately 55 and 34 times those of plasma. The high tissue to plasma ratios were maintained for at least 7 days after administration. Similarly, high tissue to plasma ratios (> 100) were found in dogs treated with a clinically relevant dose of ZD1694. These were maintained for 4 weeks in liver and kidney tissue (> 100). Total gastrointestinal concentrations of ZD1694 were approximately 10 times higher than plasma 3 days after administration, but levels were near to the limit of detection at 4 weeks. These results are consistent with extensive polyglutamation of ZD1694 within tissues in both mice and dog and provide further support for the infrequent schedule that has been used clinically. Although it has not been possible to measure individual polyglutamated forms of ZD1694, the radioimmunoassay provides a convenient means of assessing total drug levels in tissues and is currently the only method suitable for measuring the extent of drug retention in normal tissue and tumour biopsies obtained from patients treated with ZD1694.


British Journal of Cancer (1998) 77(2), 221–226
© 1998 Cancer Research Campaign

### Comparison of plasma and tissue levels of ZD1694 (Tomudex), a highly polyglutamatable quinazoline thymidylate synthase inhibitor, in preclinical models

**GW Aherne[1], E Ward[1], N Lawrence[1], D Dobinson[1], SJ Clarke[1], H Musgrove[1], F Sutcliffe[2], T Stephens[2] and AL Jackman[1]**

[1]CRC Centre for Cancer Therapeutics, Institute of Cancer Research, 15 Cotswold Road, Sutton, UK; [2]Zeneca Pharmaceuticals, Alderley Park, Macclesfield, Cheshire, UK

**Summary** ZD1694 (Tomudex, raltitrexed) is a specific quinazoline antifolate thymidylate synthase inhibitor that relies on polyglutamation for high potency. Antibodies to ZD1694 have been used to establish a sensitive radioimmunoassay as an alternative to high-performance liquid chromatography (HPLC). The radioimmunoassay is reproducible, accurate and provides a means of determining low levels of ZD1694 in plasma (< 1 nM). By virtue of the high cross-reactivity of the antibodies with polyglutamated forms of ZD1694, it is also possible to measure the total concentration of drug in tissues. Results obtained in L1210 mouse leukaemia cells and in mouse tissues were similar to those previously determined using radiolabelled drug. Pharmacokinetic studies in mice have confirmed that the compound is rapidly eliminated from the plasma and that there is a prolonged terminal elimination phase. ZD1694 was measured in plasma (0.56 ng $ml^{-1}$; 1.2 pmol $ml^{-1}$) up to 7 days after a single i.p. dose of 100 mg $kg^{-1}$ ZD1694. Liver, kidney and gut epithelium had a substantially higher level of ZD1694 immunoreactivity than plasma. For example, 24 h after a single i.p. dose at 1, 10 and 100 mg $kg^{-1}$, total drug levels in the liver were 480, 325 and 152 times higher than plasma levels respectively. In kidney and gut epithelium, total drug levels at these doses were approximately 55 and 34 times those of plasma. The high tissue to plasma ratios were maintained for at least 7 days after administration. Similarly, high tissue to plasma ratios (> 100) were found in dogs treated with a clinically relevant dose of ZD1694. These were maintained for 4 weeks in liver and kidney tissue (> 100). Total gastrointestinal concentrations of ZD1694 were approximately 10 times higher than plasma 3 days after administration, but levels were near to the limit of detection at 4 weeks. These results are consistent with extensive polyglutamation of ZD1694 within tissues in both mice and dog and provide further support for the infrequent schedule that has been used clinically. Although it has not been possible to measure individual polyglutamated forms of ZD1694, the radioimmunoassay provides a convenient means of assessing total drug levels in tissues and is currently the only method suitable for measuring the extent of drug retention in normal tissue and tumour biopsies obtained from patients treated with ZD1694.

**Keywords:** ZD1694 (Tomudex, raltitrexed); radioimmunoassay; thymidylate synthase inhibitor; polyglutamation; pharmacokinetics

Thymidylate synthase (TS; EC 2.1.1.45), which converts dUMP to thymidylate, plays an essential role in the synthesis of DNA. In recent years this enzyme has received much attention as a chemotherapeutic target (Nord and Martin, 1993; Jackman and Calvert, 1995), and several compounds are at various stages of drug development. One of these, ZD1694 (Tomudex, raltitrexed), a quinazoline antifolate (Jackman et al, 1991*a*, 1995*a*), has progressed to broad phase II (Zalcberg et al, 1995; Smith et al, 1996) and phase III clinical studies in advanced colorectal cancer (Cunningham et al, 1995). Tomudex is now available for the treatment of advanced colorectal cancer in several countries.

ZD1694 inhibits TS with a $K_i$ for the mouse enzyme of 60 nM (Jackman et al, 1991*a*), is actively transported into cells through the reduced folate carrier (RFC) and then rapidly metabolized by the enzyme folylpolyglutamate synthetase (FPGS). The polyglutamated forms of ZD1694 are more effective inhibitors of TS than the parent compound (e.g. the $K_i$ for mouse enzyme of the tetraglutamate metabolite is 1 nM) and are also retained within cells (Jackman et al, 1991*a*; Gibson et al, 1993). The prolonged inhibition of TS in intact cells (Jackman et al, 1995*b*; Aherne et al, 1996*a*), even after removal of drug from the medium, is consistent with the formation and retention of active intracellular polyglutamated species. High circulating levels of thymidine in mouse models compared with those in man complicate the anti-tumour evaluation of TS inhibitors (Jackman et al, 1984, 1995*a*), but ZD1694 was curative in DBA2 mice bearing the intramuscular L5178Y TK–/– mouse lymphoma by a single i.p. injection (10 mg $kg^{-1}$) (Jackman et al, 1991*b*). In thymidine salvage competent models, repeated treatment over 5 days with ZD1694 led to anti-tumour activity (Jackman et al, 1991*a* and *b*). Prolonged TS inhibition in the tissues and tumours of mice (presumably due to drug retention) was the basis for the once every 3 weeks schedule used in clinical studies (Jackman et al, 1995*a*; Clarke et al, 1996).

ZD1694 has previously been measured in plasma by reversed-phase HPLC (Jodrell et al, 1991). The limit of detection of this assay for plasma was 0.2 μM when a solid-phase extraction was used. In mice treated with 100 mg $kg^{-1}$, plasma levels could be followed for up to 10 h. The analysis showed that the drug is eliminated rapidly from the plasma (with a half-life of ~ 30 min), and there was also a further prolonged phase of elimination (~6.5 h). A

*Received 14 February 1997*
*Revised 24 June 1997*
*Accepted 2 July 1997*

*Correspondence to:* GW Aherne long terminal plasma elimination phase of ZD1694 has also been measured in man using $^{14}C$-radiolabelled compound (Judson et al, 1996). Drug was measured in the plasma (1–10 ng ml$^{-1}$) for up to 4 weeks after drug administration, giving a third phase half-life of 257 h. In a phase I trial of the drug (Clarke et al, 1996), no correlations between plasma pharmacokinetic parameters ($C_{max}$ or AUC) and clinical outcome (toxicity or response) were found.

Because of the highly polyglutamatable nature of ZD1694, total concentration of drug (parent compound and polyglutamates) in tissues and tumour is likely to be a more important determinant of clinical outcome than plasma drug levels. Polyglutamated forms of the quinazoline antifolates have previously been measured in vitro using radiolabelled drugs. Separation of polyglutamated metabolites by HPLC was followed by quantitation in fractions using radiochemical analysis (Jackman et al, 1991*c*; Gibson et al, 1993) or by measuring inhibition of TS (Sikora et al, 1988). In preliminary experiments, direct evidence of polyglutamation in tissues has been obtained by administration of radiolabelled drug to mice (Jackman et al, 1995*a*). Total drug concentrations in liver, kidney and intestinal epithelium were 30- to 100-fold higher than in the plasma 24 h after a single injection of tritiated ZD1694 (5 mg kg$^{-1}$), and most of the radioactivity (~80%) in the tissues was present as polyglutamates. No polyglutamates were found in mouse plasma.

However, the use of radiolabelled ZD1694 is not suited to extended pharmacokinetic studies or to any clinical studies on tissue and tumour drug retention. As part of our efforts to provide further evidence for the importance of polyglutamation in the pharmacology and activity of Tomudex, a sensitive radioimmunoassay has been used to compare the total drug concentrations in mouse and dog tissues with those in plasma. The dog was chosen as an additional model, because, unlike mice, plasma levels of thymidine are low and this species is much more sensitive to the effects of TS inhibitors than rodents. Dog toxicity studies were found to predict well for the maximum-tolerated dose of ZD1694 in clinical trials (data on file, Zeneca Pharmaceuticals). Thus this animal model would be expected to give a good indication of ZD1694 concentrations in human tissues at a relevant therapeutic dose.

### METHODS

Sheep antiserum to ZD1694 (S37B14) was provided by Zeneca Pharmaceuticals and was stored at 4°C with the addition of 0.1% sodium azide. Tritiated ZD1694 (19.1 Ci mmol$^{-1}$) was prepared by Cambridge Research Biochemicals and supplied by Zeneca Pharmaceuticals. Acetonitrile was from Fisons, Dextran T70 from Pharmacia and all other chemicals from Sigma. Solutions were prepared with deionized water. ZD1694 was supplied by Zeneca Pharmaceuticals, and the synthesis of the polyglutamated forms of ZD1694 and its analogues has been previously described (Hughes et al, 1990; Bisset et al, 1992; Bavetsias et al, 1993).

Stock solutions (10 mM) were prepared in 0.15 M sodium bicarbonate and stored at –20°C. For the mouse studies, the drug was formulated in 0.05 M sodium bicarbonate and the pH was adjusted to 8.5–9.0.

**Table 1** Cross-reaction of S37B14 antiserum with ZD1694 polyglutamates and natural folates

| Compound | % |
|---|---|
| ZD1694 | 100 |
| Diglutamate | 90 |
| Triglutamate | 92 |
| Tetraglutamate | 110 |
| Pentaglutamate | 110 |
| Hexaglutamate | 100 |
| Leucovorin | < 0.01 |
| Methyl-THFA | < 0.01 |

The cross-reactants were incubated at various concentrations with the ZD1694 antiserum and radiolabelled ZD1694, as described in the methods. Percentage cross-reactivity is the relative amount of ZD1694 and cross-reactant required to inhibit antibody binding of radiolabel by 50%.

### Radioimmunoassay

The assay diluent was 0.05 M phosphate buffer pH 7.4, containing 1 g l$^{-1}$ gelatin and 6 g l$^{-1}$ sodium chloride (PBSG). Initially, all dilutions and dispensing of standards and samples were made with a Dilutrend diluter, but a Multiprobe (Canberra Packard) automated liquid-handling system was later programmed to perform these steps in the assay. A working standard solution of ZD1694 (100 μg ml$^{-1}$) was stored in aliquots at –20°C and diluted in PBSG for each assay to cover the range of the standard curve. Samples were assayed at three appropriate dilutions to ensure that the results of at least two dilutions fell on the linear portion of the standard curve, and samples were reassayed at different dilutions if necessary. The antiserum was diluted in PBSG so that approximately 40% of total radioactivity was bound (Bo) to antibody. This dilution was previously determined from an antiserum dilution curve and was 1:300 to 1:500 depending on the batch of radiolabel used. [$^3$H]ZD1694 was also diluted (from a sub-stock stored at –20°C) in PBSG so that approximately 3 pmol (in 100 μl) was added to each assay tube.

Diluted standards and samples (0.1 ml) were added in duplicate to numbered LP3 tubes (Luckham) or to deep-well blocks (Beckman) with 0.3 ml of assay diluent, 0.1 ml of diluted antiserum and 0.1 ml of diluted label, mixed and left to stand in iced water for 1 h. Each assay included total counts and non-specific-binding tubes, containing only the radiolabel and buffer, and zero-standard-binding tubes (Bo), which contained only radiolabel and antiserum. The antibody-bound radiolabel was separated from unbound radiolabel by the addition of ice-cold dextran-coated charcoal [2.5% (w/v) activated charcoal (Sigma) coated with 0.25% (w/v) Dextran T-70 (Pharmacia)] to all but the total-counts tubes for 10 min. After centrifugation at 1000 ***g*** for 10 min at 4°C, 0.5 ml aliquots of supernatant were taken from each assay tube for scintillation counting in 4.0 ml of Ultima gold scintillant (Canberra-Packard). The ZD1694 concentrations in the samples were calculated from the standard curve with a data reduction programme that used a four-parameter logistic plot (RiaSmart, Canberra-Packard). The radioimmunoassays were carried out at the Institute of Cancer Research.

### Intracellular drug levels

L1210 mouse leukaemia cells (2–3 × 10$^6$) and an acquired resistant variant of the cell line (L1210R:$^{D1694}$; 6–8×10$^6$ cells), which has an impaired ability to polyglutamate ZD1694 (Jackman et al, 1995*c*), were exposed to ZD1694 (0.1 μM and 1 μM) for 4 or 24 h and, after harvesting and washing as described previously (Gibson et al, 1993; Jackman et al, 1995*c*), were resuspended in 0.5 ml of

© Cancer Research Campaign 1998

**Table 2** Intracellular concentrations of ZD1694 in L1210 mouse leukaemia cells and an acquired resistant variant with impaired ability to polyglutamate antifolates

| Cell line | ZD1694 exposure | Intracellular ZD1694 (μM) |
|---|---|---|
| L1210 | 0.1 μM, 4 h | 1.04 ± 0.25 ($n$ = 4) |
| | 0.1 μM, 24 h | 4.65 (4.9/4.4) |
| L1210:R[D1694] | 0.1 μM, 24 h | ND |
| L1210 | 1 μM, 4 h | 5.98 ± 0.56 ($n$ = 4) |
| | 1 μM, 24 h | 17.2 (19.7/14.7) |
| L1210:R[D1694] | 1 μM, 24 h | 0.12 (0.14/0.10) |

Each extract was assayed in duplicate at three dilutions. Intracellular concentrations were based on a L1210 cell volume of $0.63 \times 10^{-12}$ l. ND, none detected. The limit of detection for 6–8 × $10^6$ cells is approximately 0.05 μM intracellular concentration.

**Figure 1** The disappearance of ZD 1694 (5 mg kg$^{-1}$ i.p.) from the plasma of female DBA2 mice ($n$ = 3). Error bars are ± s.d. and are within the symbol for the 1-h time point. The 2- and 24-h time points are the means of two animals. Predicted concentrations (*) were obtained using a non-compartmental model (PCNonlin)

PBSG and frozen. After thawing, the cells were sonicated for 3 × 30 s, the supernatant clarified by centrifugation (1000 *g* for 10 min) and then assayed at three dilutions.

### Mouse pharmacokinetic studies

Female DBA2 mice aged between 7 and 9 weeks were treated in groups of three with a single i.p. injection (0.1 ml per 10 g) of ZD1694 (5 mg kg$^{-1}$). Blood was obtained by cardiac puncture under anaesthetic after 0.5, 1, 2, 4, 9 and 24 h. Mice were also treated i.p. at 1, 10 and 100 mg kg$^{-1}$ ZD1694; blood, liver and kidney were obtained 24 h later and, additionally, for animals treated with 100 mg kg$^{-1}$, 2, 4 and 7 days later to assess tissue retention of ZD1694. Plasma was immediately separated from the blood by microcentrifugation and stored at –20°C until assayed. Liver and kidney samples were immediately frozen on solid carbon dioxide. Gut epithelium samples were obtained 1 day after drug treatment. A section (5–15 cm below the stomach) of upper small intestine was flushed through with cold 0.1 M Tris-acetate buffer pH 10 and slit open; the epithelium was scraped into a weighed glass pot containing 0.5 ml of Tris buffer and stored frozen.

Plasma samples were assayed directly in the radioimmunoassay at three dilutions depending on the dose and time since administration. Liver and kidney tissues were thawed, weighed and homogenized with two volumes 0.1 M Tris-acetate buffer pH 10 and aliquots of homogenates (0.5 ml) were treated with an equal volume of acetonitrile. The weighed gut-scrape samples were sonicated for 30 s and also extracted with an equal volume of acetonitrile. After centrifugation to remove particulate matter (8000 *g* for 10 min), the tissue extracts were aspirated and diluted with PBSG before radioimmunoassay. Again, three dilutions in duplicate were assayed.

### Pharmacokinetic studies in dogs

ZD1694 (0.2 mg kg$^{-1}$) was administered by single intravenous bolus injection to pairs of Alderley Park Beagle dogs (one female and one male dog). Blood was obtained from one pair 4 h, 24 h, 3,

**Table 3** Plasma ZD1694 concentrations and total ZD1694 immunoreactivity in tissues 1–7 days after a single i.p. administration of ZD1694 in female DBA2 mice

| Dose (mg kg)$^{-1}$ | Day | Plasma (ng ml)$^{-1}$ | Tissue (nmol g$^{-1}$) ZD1694 equivalents (tissue–plasma ratio) | | |
|---|---|---|---|---|---|
| | | | Liver | Kidney | Gut epithelium |
| 1 | 1 | 2.26 ± 1.1<br>(4.75 ± 2.3)[a] | 2.28 ± 0.8<br>(480) | 0.32 ± 0.1<br>(67) | 0.17 ± 0.05<br>(36) |
| 10 | 1 | 3.42 ± 1.0<br>(7.18 ± 2.1)[a] | 2.33 ± 0.6<br>(325) | 0.38 ± 0.08<br>(53) | 0.36 (0.38 : 0.33)<br>(50) |
| 100 | 1 | 14.6 ± 7.3[b]<br>(30.67 ± 15.3)[a] | 4.65 ± 2.8[b]<br>(152) | 1.40 ± 0.2[b]<br>(46) | 0.53 ± 0.09<br>(17) |
| | 2 | 4.0 ± 0.6<br>(8.40 ± 1.3)[a] | 0.86 ± 0.3<br>(102) | 0.46 ± 0.1<br>(55) | ND |
| | 4 | 1.14 ± 0.4<br>(2.39 ± 0.8)[a] | 0.41 ± 0.12<br>(172) | 0.22 ± 0.06<br>(92) | ND |
| | 7 | 0.56 ± 0.1<br>(1.18 ± 0.2)[a] | 0.15 ± 0.07<br>(127) | 0.10 ± 0.02<br>(85) | ND |

[a]Values as pmol ml$^{-1}$. [b]Results from two experiments. Values are the mean ± s.d. of three animals per group.

© Cancer Research Campaign 1998

7, 14, 21 and 28 days after dosing. On day 28, the animals were sacrificed and liver, kidney and gastrointestinal tissues (8-inch sections of duodenum, jejunum, ileum and colon) were obtained. The dogs from the other two pairs were bled, and tissues were harvested 24 h and 72 h after dosing. One dog was treated with saline and used to provide control samples. Plasma, liver (2-g samples) and kidney (2-g slices from a kidney cut tangentially to include cortex and medulla) were immediately frozen on Cardice and stored at –70°C until processed, as described for mouse tissues. The gastrointestinal samples were placed on ice and cut longitudinally and then washed with ice-cold saline to remove the contents; the epithelium was scraped with a spatula and placed in preweighed tubes and frozen. This work was carried out in the laboratories of Zeneca Pharmaceuticals.

### RESULTS

### Radioimmunoassay performance

The ZD1694 standard curve ranged from 0.1 to 10 ng $ml^{-1}$ (0.21–21 nM) and the mean dose at 50% inhibition of binding was 0.7 ± 0.05 ng $ml^{-1}$ (1.47 nM). The sensitivity of the curve as determined by a 3-s.d. fall in binding from the Bo value was 0.1 ng $ml^{-1}$. To eliminate minor matrix effects, plasma samples were diluted at least 1:2, giving a limit of detection of 0.2 ng $ml^{-1}$ (0.42 pmol $ml^{-1}$). The recovery of ZD1694 added to drug-free plasma was complete over a concentration range of 1–500 ng $ml^{-1}$ (96.4 ± 8.7%). Within and between assay variation was 14.0 ± 1.5 ng $ml^{-1}$, CV 10.7%, and 14.7 ± 1.9 ng $ml^{-1}$, CV 12.9%, respectively.

The cross-reaction of the antiserum with reduced folates and the polyglutamated forms of the drug is shown in Table 1. As expected the polyglutamated metabolites are recognized to essentially the same extent as the parent compound. This cross-reaction has been exploited to measure total ZD1694 immunoreactivity in tissues. To minimize effects of acetonitrile extracts on antibody binding, it was necessary to dilute extracts at least 10–40 times before assay, depending on the tissue assayed. Thus, taking into account all the dilutions involved in the procedure, the limit of detection for liver, kidney and gut epithelium extracts were 0.06, 0.03 and 0.015 nmol $g^{-1}$, respectively, although, in practice, extracts were normally diluted at least 50 times before assay so that antibody binding fell on the linear portion of the standard curve. Even with these high-extract dilutions, it was possible to measure ZD1694 for prolonged periods after drug administration (see later). The recovery of ZD1694 (105–1050 nM) added to tissue homogenates was 113.3 ± 18.6%, and mean interassay variation for tissue measurement was 11.9%. Results obtained between experiments were similar. For example, mice were treated on three occasions with 100 mg $kg^{-1}$ ZD1694, and results for liver extracts obtained 6 days later were not significantly different (0.73 ± 0.18, 0.67 ± 0.42 and 0.58 ± 0.18 nmol $g^{-1}$). The tissue levels of ZD1694 obtained by radioimmunoassay were similar to those measured previously using radioactive drug. At 24 h after a single 5 mg $kg^{-1}$ i.p. injection of ZD1694, total drug liver levels measured by radioimmunoassay were not significantly different (1.29 ± 0.27 nmol $g^{-1}$ ($n = 4$) ($P > 0.25$) from those measured in radioactive experiments (0.93 ± 0.5 nmol $g^{-1}$ ($n = 3$) (Jackman et al, 1995*a*).

### Intracellular ZD1694

As part of the validation of the radioimmunoassay for the measurement of total concentrations of ZD1694 (i.e. drug plus polyglutamated forms), results obtained on extracts of cells grown in the presence of drug were compared with those previously obtained by radioassay after exposure to radioactive drug. The intracellular concentration of ZD1694 in L1210 cells exposed to the drug (0.1 μM) for 4 h was 1.04 ± 0.25 μM compared with 2.9 ± 1.2 μM determined by radioassay. At 24 h the intracellular concentration was 4.65 μM compared with 3.8 μM by radioassay (Jackman et al, 1995*c*). No immunoreactivity was measurable in the L1210:$R^{D1694}$ cells after a 24-h exposure to 0.1 μM. At 1 μM drug exposure, immunoreactivity was just measurable (0.12 μM) in the resistant cells, the parent line accumulating approximately 150 times more drug at this dose (Table 2). For the resistant line, the radioassay estimated a level of total drug ZD1694 of 0.44 μM after 24 h.

**Figure 2** Total ZD1694 concentrations in the plasma (x), liver (♦), kidney (▲), duodenum (X), ileum (▼), jejunum (●) and colon (■) of dogs treated with 0.2 mg $kg^{-1}$ ZD1694 (single i.v. bolus). Results are the means of two dogs, *except at 24 and 72 h at which $n = 4$ (mean ± s.d.). In gastrointestinal epithelium, concentrations were at the limit of detection (0.015 nmol $g^{-1}$) by day 28

### ZD1694 plasma and tissue concentrations in DBA2 mice

The disappearance of ZD1694 from the plasma of DBA2 mice given 5 mg $kg^{-1}$ i.p. is shown in Figure 1. ZD1694 rapidly disappeared from the plasma with an elimination phase of approximately 2.7 h (Non-compartmental model; PCnonlin) and drug was still measurable at 24 h. Plasma and tissue levels of ZD1694 24 h after 1, 10 and 100 mg $kg^{-1}$ i.p. doses are shown in Table 3. Plasma drug concentrations increased with dose and there was evidence of a prolonged terminal elimination phase in mice treated with 100 mg $kg^{-1}$ ZD1694. By 7 days, plasma concentrations of ZD1694 were 0.56 ± 0.1 ng $ml^{-1}$ (1.18 ± 0.2 pmol $ml^{-1}$). Total ZD1694 immunoreactivity in tissues also declined with time but, at all doses and times studied, exceeded those of plasma (liver at least 100-fold, kidney at least 46-fold and gut epithelium at least 17-fold).

### ZD1694 plasma and tissue concentrations in dogs

The total drug concentrations in the plasma and tissues from dogs treated with ZD1694 are shown in Figure 2. As plasma samples were obtained at only two time points during the first day, the early

© Cancer Research Campaign 1998

plasma distribution and elimination drug kinetics have not been defined. However, it is apparent that drug elimination within the first 24 h is rapid and that a long terminal elimination phase is present, plasma concentrations declining to approximately 0.4 ng $ml^{-1}$; 0.85 pmol $ml^{-1}$ 28 days after drug administration. Total ZD1694 immunoreactivity in liver and kidney was at least 100 times that in plasma throughout the 28 days of the study. In the gastrointestinal tissues (duodenum, jejunum, ileum, colon), total immunoreactivity was approximately 10 times that of plasma 24 and 72 h after administration but had declined so that at 28 days drug concentrations were at or near the limit of detection of the assay (0.015 nmol $ml^{-1}$). There were no consistent differences between female and male dogs.

### DISCUSSION

Because of the high potency of ZD1694 and its rapid elimination from plasma (Jackman et al, 1991*a*; Jodrell et al, 1991), a highly sensitive assay is required for extended pharmacokinetic studies; radioimmunoassay offers an attractive alternative to HPLC. A sheep antiserum to ZD1694 has been used for the radioimmunoassay of plasma samples and it is specific and sensitive with consistent day-to-day performance. As there is as yet no evidence of plasma and urinary metabolites of ZD1694 (Jodrell et al, 1991; Judson et al, 1996), plasma drug measurements made with the antiserum can be considered to be specific for ZD1694. The remarkable ability of antisera to distinguish between antifolates and natural folates has been shown before (Levine and Powers, 1974; Aherne et al, 1977) and the low cross-reaction of the ZD1694 antiserum with reduced folates was therefore not surprising. From previous work, it is also to be expected (Anzai et al, 1987) that polyglutamated forms of antifolates will be recognized to a similar extent as the monoglutamate.

The plasma pharmacokinetics of ZD1694 (5 mg $kg^{-1}$ i.p.) in mice have been measured using the radioimmunoassay. The calculated half-life is similar to previous estimates determined by HPLC but after a much higher dose (100 mg $kg^{-1}$ i.p.) (Jodrell et al, 1991). Further, evidence of a slower phase of elimination has been obtained in this study and, after 100 mg $kg^{-1}$, plasma concentrations of ZD1694 were still measurable at least 7 days after drug administration. Additionally, in dogs, ZD1694 was still present in plasma 28 days after administration, which is consistent with the long terminal half-life reported in patients (Judson et al, 1996). The importance of this prolonged period of plasma ZD1694 exposure remains to be established.

At the three doses used in this study, total ZD1694 immunoreactivity in mouse liver and kidney were > 100-fold and ~55-fold higher, respectively, at 24 h than plasma concentrations of ZD1694 (Table 3). At 100 mg $kg^{-1}$, this ratio remained high, at least until 7 days after drug administration. High tissue to plasma ratios (~34-fold) were also observed in gut epithelium at 24 h. Similarly, in the dog, there was considerable accumulation of immunoreactive ZD1694 in liver and kidney (100–150 times) compared with plasma, although in the gastrointestinal epithelium the tissue to plasma ratio was lower (approximately 10). The loss of drug in these tissues by day 28 could be due to cell death, hydrolysis and subsequent efflux of ZD1694 monoglutamate or to dilution of ZD1694 drug species by cell division. These observations are consistent with (but do not prove) the efficient uptake and retention of ZD1694 as polyglutamated species within the tissues.

Polyglutamation plays an important role in the activity of several antifolates. Their measurement in tissue culture cells has been achieved previously after incubation with radiolabelled compounds followed by extraction and HPLC separation (Jolivet et al, 1982; Sikora et al, 1988; Pizzorno et al, 1989; Jackman et al, 1991*c*; Gibson et al, 1993). Similar methodology has been used to measure the polyglutamated forms of methotrexate in isolated human leukaemic blast cells (Goker et al, 1993). Methotrexate polyglutamates have been determined in human tissue samples after high-dose therapy by using HPLC with subsequent detection in fractions using radioimmunoassay (Anzai et al, 1987) or by using the radioligand binding assay (Samuels et al, 1984). Total methotrexate has also been measured by radioimmunoassay as a measure of polyglutamation in erythrocytes from patient treated with high-dose therapy (Schalhorn et al, 1982).

A similar approach has been used in the present study. Our initial experiments showed that the radioimmunoassay was not sufficiently sensitive to determine the amounts of individual polyglutamates in fractions obtained from HPLC. As the polyglutamates of ZD1694 cross-react (on a molar basis) to essentially the same extent as the parent drug, experiments were carried out to determine if this cross-reactivity could be used to measure total drug levels (presumed to be ZD1694 parent drug and polyglutamates). Firstly, total immunoreactivity measured in vitro and in vivo compared well with measurements of total drug determined using radiolabelled ZD1694. Secondly, it has previously been shown that at 24 h the majority (80%) of radiolabelled ZD1694 in tissues was present in the polyglutamated forms (Jackman et al, 1995*b*), and the high tissue to plasma ratios described here are also presumed to represent polyglutamated forms of this antifolate. Thirdly, the high tissue to plasma ratios observed for ZD1694 in both mouse and dog tissues are in contrast to those observed with non-polyglutamated quinazoline antifolates. For example, liver levels of CB30900 were only sixfold higher than plasma levels, which were similar to kidney concentrations (Walton et al, 1996). Similarly, low tissue to plasma ratios were also observed for ZD9331, a non-polyglutamated TS inhibitor in clinical study (Aherne et al, 1996*b*). While this assay gives no indication of which polyglutamates are present, the duration and extent of drug retention in tissues, thought to be primarily due to polyglutamation, can be obtained. The concentration and retention of ZD1694 in tissues provides further justification for the infrequent bolus administration being used clinically.

In summary, we have described a convenient and sensitive radioimmunoassay for determining the concentration of ZD1694 in biological samples. As the antiserum used cross-reacts with the polyglutamated forms of ZD1694, total ZD1694 immunoreactivity in tissues is thought to represent parent monoglutamate and polyglutamates. The initial rapid elimination of ZD1694 from plasma in mice has been confirmed as well as the persistence of low plasma drug levels described in mice and dogs. Total ZD1694 in normal tissues (liver, kidney and gut epithelium) exceeded those of plasma, the high ratio being maintained in liver and kidney for at least 7 days in mice and 28 days in dogs after the administration of a single dose. The availability of this radioimmunoassay presently represents the most convenient means by which total drug levels in tissues and tumours can be obtained preclinically, and it is presently being used to provide information on the pharmacokinetic/pharmacodynamic relationships of ZD1694 C Hardcastle et al, 1997) in addition to the effects of potential rescue agents, such as leucovorin (Jackman et al, 1995*d*). For clinical biopsy material

© Cancer Research Campaign 1998 

(in which the amount of tissue available for assay is small), the radioimmunoassay represents the only method that is currently available for determining drug levels (Farrugia et al, 1997).

### ACKNOWLEDGEMENTS

The authors would like to thank Dr D Farrugia and Mrs Anthea Hardcastle for their help and interest in this work and Miss D Hughes for technical assistance. This work was supported by the Cancer Research Campaign.

### REFERENCES

Aherne GW, Piall EM and Marks V (1977) Development and application of a radioimmunoassay for methotrexate. *Br J Cancer* **36**: 608–617

Aherne GW, Hardcastle A, Raynaud F and Jackman AL (1996*a*) Immunoreactive dUMP and TTP pools as an index of thymidylate synthase inhibition; effect of Tomudex (ZD1694) and a non-polyglutamated quinazoline antifolate (CB30900) in L1210 mouse leukaemia cells. *Biochem Pharmacol* **51**: 1293–1301

Aherne GW, Ward E, Dobinson D, Hardcastle A and Jackman AL (1996*b*) Pharmacokinetics of a bolus injection of ZD9331, a non-polyglutamated thymidylate synthase (TS) inhibitor. *Proc Am Assoc Cancer Res* **37**: 382

Anzai T, Jaffe N and Wang Y-E (1987) Separation and identification of methotrexate and its metabolites, 7-hydroxymethotrexate and polyglutamates, in human tissues by reversed-phase high-performance liquid chromatography coupled with radioimmunoassay. *J Chromatography* **415**: 445–449

Bavetsias V, Jackman AL, Thornton TJ, Pawelczak K, Boyle FT and Bisset GMF (1993) Quinazoline antifolates inhibiting thymidylate synthase; synthesis of γ-peptide and amide analogues of 2-desamino-2-methyl-N[10]-propargyl-5,8-dideazafolic acid (ICI198583). In *Chemistry and Biology of Pteridines and Folates*, Vol. 338, Ayling JE, Nair MG and Baugh CM. (eds), pp. 593–596. Plenum Press: New York

Bisset GMF, Pawelczak K, Jackman AL, Calvert AH and Hughes LR (1992) The synthesis and thymidylate synthase inhibitory activity of the poly-γ-glutamyl) conjugates of ICI D1694 and other quinazoline antifolates. *J Med Chem* **35**: 859–866

Clarke SJ, Hanwell J, deBoer M, Planting A, Verweij J, Walker M, Smith R, Jackman AL, Hughes LR, Harrap KR, Kennealy GT and Judson IR (1996) Phase 1 trial of ZD1694 ("Tomudex"), a new folate based thymidylate synthase inhibitor, in patients with solid tumours. *J Clin Oncol* **14**: 1495–1503

Cunningham D, Zalcberg JR, Rath U, Olver I, van Cutsem E, Svensson C, Seitz JF, Harper P, Kerr D, Perez-Manga G, Azab M, Seymore L, Lowery K and the Tomudex Colorectal Cancer Study Group (1995) Tomudex (ZD1694): results of a randomised trial in advanced colorectal cancer demonstrate efficacy and reduced mucositis and leucopenia. *Eur J Cancer* **31A**: 1945–1953

Farrugia D, Cunningham D, Danenberg P, Danenberg K, Metzger R, Mitchell F, MacVicar D, McCarthy K, Aherne GW, Norman A and Jackman AL (1997) A pharmacodynamic (PD) study of the thymidylate synthase (TS) inhibitor Tomudex in advanced colorectal cancer (CRC). *Proc Am Assoc Cancer Res* **38**: 615

Gibson W, Bisset GMF, Marsham PR, Kelland LR, Judson IR and Jackman AL (1993) The measurement of polyglutamate metabolites of the thymidylate synthase inhibitor, ICI D1694, in mouse and human cultured cells. *Biochem Pharmacol* **45**: 863–869

Goker E, Lin JT, Trippett T, Elisseyeff Y, Tong WP, Niedzwiecki D, Tan C, Steinherz P, Schweitzer BI and Bertino JR (1993) Decreased polyglutamylation of methotrexate in acute lymphoblastic leukemia blasts in adults compared to children with this disease. *Leukemia* **7**: 1000–1004

Hardcastle A, Dobinson D, Farrugia D, Jackman AL and Aherne GW (1997) In vivo pharmacokinetic and pharmacodynamic studies of two specific thymidylate synthase (TS) inhibitors. *Br J Cancer* **75** (suppl. 1): 25

Hughes LR, Jackman AL, Oldfield J, Smith RC, Burrows KD, Marsham PR, Bishop JAM, Jones TR, O'Connor BM and Calvert AH (1990) Quinazoline antifolate thymidylate synthase inhibitors: alkyl, substituted alkyl and aryl substituents in the C2 position. *J Med Chem* **33**: 3060–3067

Jackman AL and Calvert AH (1995) Folate-based thymidylate synthase inhibitors as anticancer drugs. *Ann Oncol* **6**: 871–881

Jackman AL, Taylor GA, Calvert AH and Harrap KR (1984) Modulation of antimetabolite effects: effects of thymidine on the efficacy of the quinazoline-based thymidylate synthetase inhibitor, CB3717. *Biochem Pharmacol* **33**: 3269–3275

Jackman AL, Taylor GA, Gibson W, Kimbell R, Brown M, Calvert AH, Judson IR and Hughes LR (1991*a*) ICI D1694, a quinazoline antifolate thymidylate synthase inhibitor that is a potent inhibitor of L1210 tumor cell growth *in vitro* and *in vivo*: new agent for clinical study. *Cancer Res* **51**: 5579–5586

Jackman AL, Jodrell DI, Gibson, W and Stephens TC (1991*b*) ICI D1694, an inhibitor of thymidylate synthase for clinical study. In *Purine and Purimidine Metabolism in Man*, Vol. VII, part A, pp. 19–23. Plenum Press: New York

Jackman AL, Newell DR, Gibson W, Jodrell DI, Taylor GA, Bishop JA, Hughes LR and Calvert AH (1991*c*) The biochemical pharmacology of the thymidylate synthase inhibitor, 2-desamino-2-methyl-N[10]-propargyl-5,8-dideazafolic acid (ICI 198583). *Biochem Pharmacol* **42**: 1885–1895

Jackman AL, Farrugia DC, Gibson W, Kimbell R and Harrap KR (1995*a*) ZD1694 (Tomudex): a new thymidylate synthase inhibitor with activity in colorectal cancer. *Eur J Cancer* **31A**: 1277–1282

Jackman AL, Kimbell R, Brown M, Brunton L and Boyle FT (1995*b*) Quinazoline thymidylate synthase inhibitors: methods for assessing the contribution of polyglutamation to their *in vitro* activity. *Anti-Cancer Drug Design* **10**: 555–572

Jackman AL, Kelland LR, Kimbell R, Brown M, Gibson W, Aherne GW, Hardcastle A and Boyle FT (1995*c*) Mechanisms of acquired resistance to the quinazoline thymidylate synthase inhibitor ZD1694 (Tomudex) in one mouse and three human cell lines. *Br J Cancer* **71**: 914–924

Jackman AL, Farrugia DC, Clarke SJ, Aherne GW, Boyle FT, Seymore L, Azab M and Kennealey G (1995*d*) Delayed rescue of ZD1694 toxicity in Balb/c mice with thymidine (dThd) or Leucovorin (LV). *Proc Am Assoc Cancer Res* **36**: 377

Jodrell DI, Newell DR, Gibson W, Hughes LR and Calvert AH (1991) The pharmacokinetics of the quinazoline antifolate ICI D1694. *Cancer Chemother Pharmacol* **28**: 331–338

Jolivet J, Schilsky RL, Bailey BD, Drake JC and Chabner BA (1982) Synthesis, retention and biological activity of methotrexate polyglutamates in cultured human breast cancer cells. *J Clin Invest* **70**: 351–360

Judson IR, Aherne GW, Maughan T, Cunningham D, Hanwell J, Berry C and Walker M (1996) Pharmacokinetic studies with Tomudex (ZD1694). *Ann Oncol* **7** (suppl. 1): 88

Levine L and Powers E (1974) Radioimmunoassay for methotrexate. *Res Commun Chem Path Pharmacol* **9**: 543–554

Nord DN and Martin DS (1993) Enhancement of thymidylate synthase inhibition. *Curr Opinion Oncol* **5**: 1017–1022

Pizzorno G, Chang Y-M, McGuire JJ and Bertino JR (1989) Inherent resistance of squamous carcinoma cell lines to methotrexate as a result of decreased polyglutamation of this drug. *Cancer Res* **49**: 5275–5280

Samuels LL, Feinberg A, Moccio DM, Sirotnak FM and Rosen G (1984) Detection by high-performance liquid chromatography of methotrexate and its metabolites in tumour tissue from osteosarcoma patients treated with high-dose methotrexate/leucovorin rescue. *Biochem Pharmacol* **33**: 2711–2714

Schalhorn A, Sauer H, Wilmanns W and Stupp-Poutot G (1982) Pharmacokinetics of erythrocyte methotrexate after high-dose methotrexate. *Cancer Chemother Pharmacol* **9**: 65–69

Sikora E, Newell DR, Jackman AL, Simmonds AJ, Jones TR and Calvert AH (1988) Development of an assay for the estimation of *N*[10]-propargyl-5,8-dideazafolic acid polyglutamates in tumour cells. *Anal Biochem* **172**: 344–355

Smith I, Jones A, Spielmann M, Namer M, Green MD, Bonneterre J, Wander HE, Hatschek T, Wilking N, Zalcberg J, Spiers J and Seymore L (1996) A phase II study in advanced breast cancer: ZD1694 (Tomudex) a novel direct and specific thymidylate synthase inhibitor. *Br J Cancer* **74**: 479–481

Walton MI, Gibson W, Aherne GW, Lawrence N, Stephens TC, Smith MN and Jackman AL (1996) Preclinical pharmacology of CB30900, a novel dipeptide inhibitor of thymidylate synthase, in mice. *J Exp Ther Pharmacol* **277**: 909–916

Zalcberg J, Cunningham D, Green M, Francois E, Van Cutsem E, Schornagel J, Adenis A, Seymore L and Azab M (1995) The final results of a large Phase II study of the potent thymidylate synthase (TS) inhibitor Tomudex (ZD1694) in advanced colorectal cancer. *Proc Am Soc Cancer Res* **4**: 204

© Cancer Research Campaign 1998


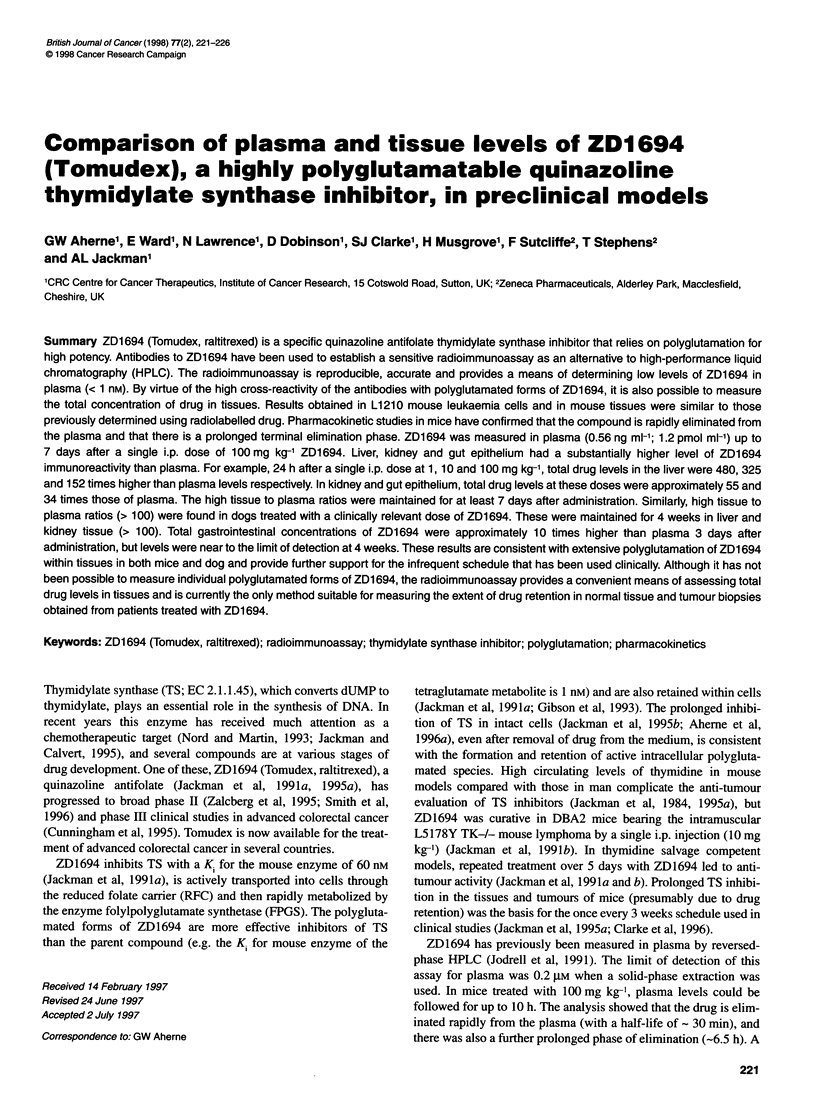

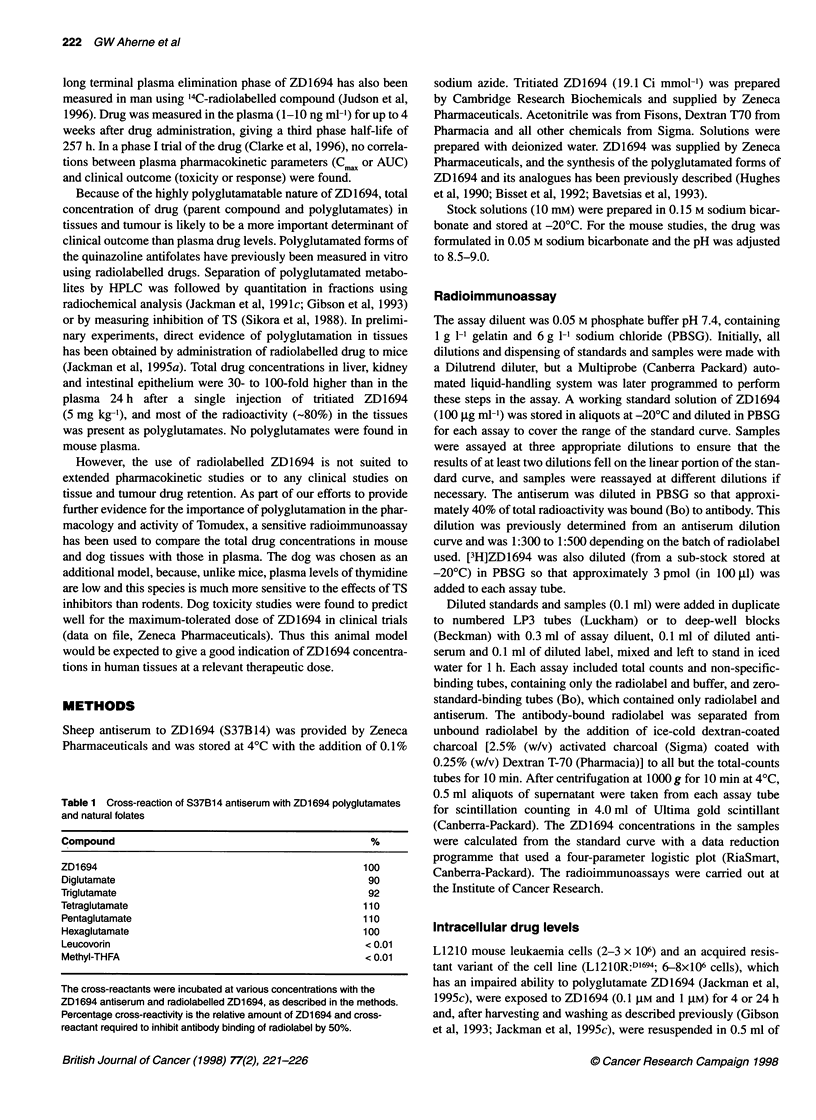

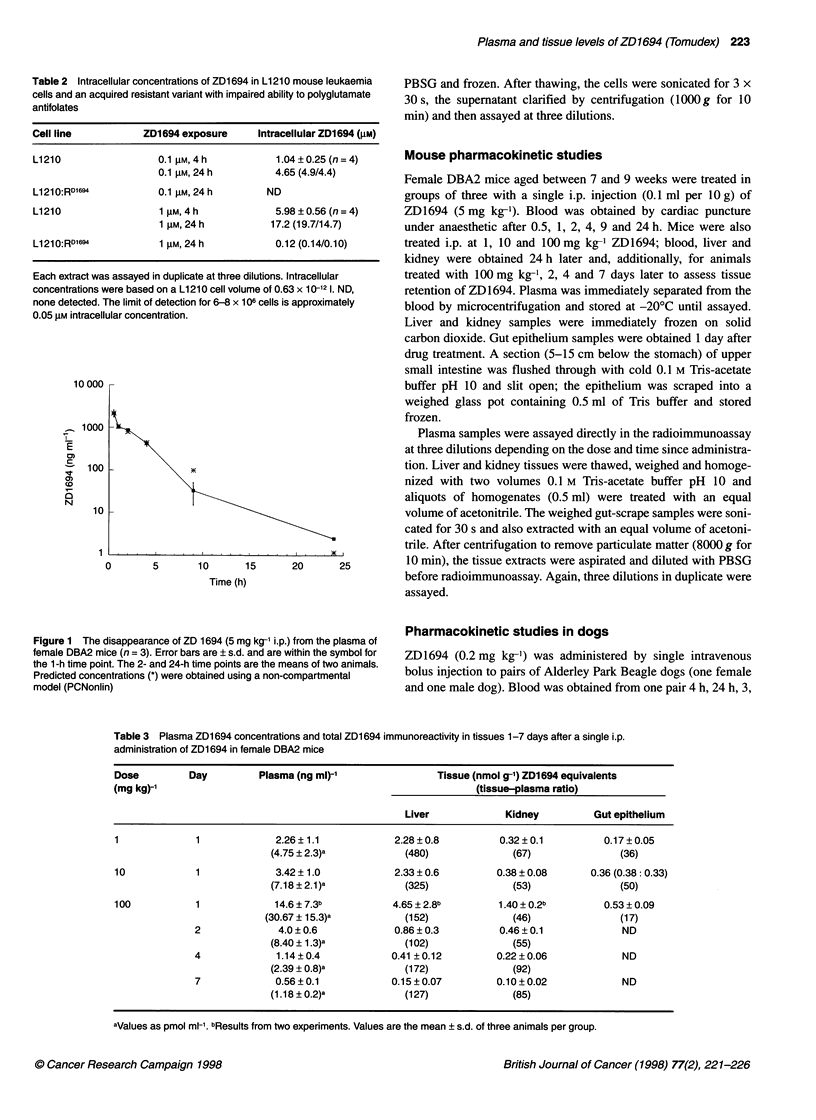

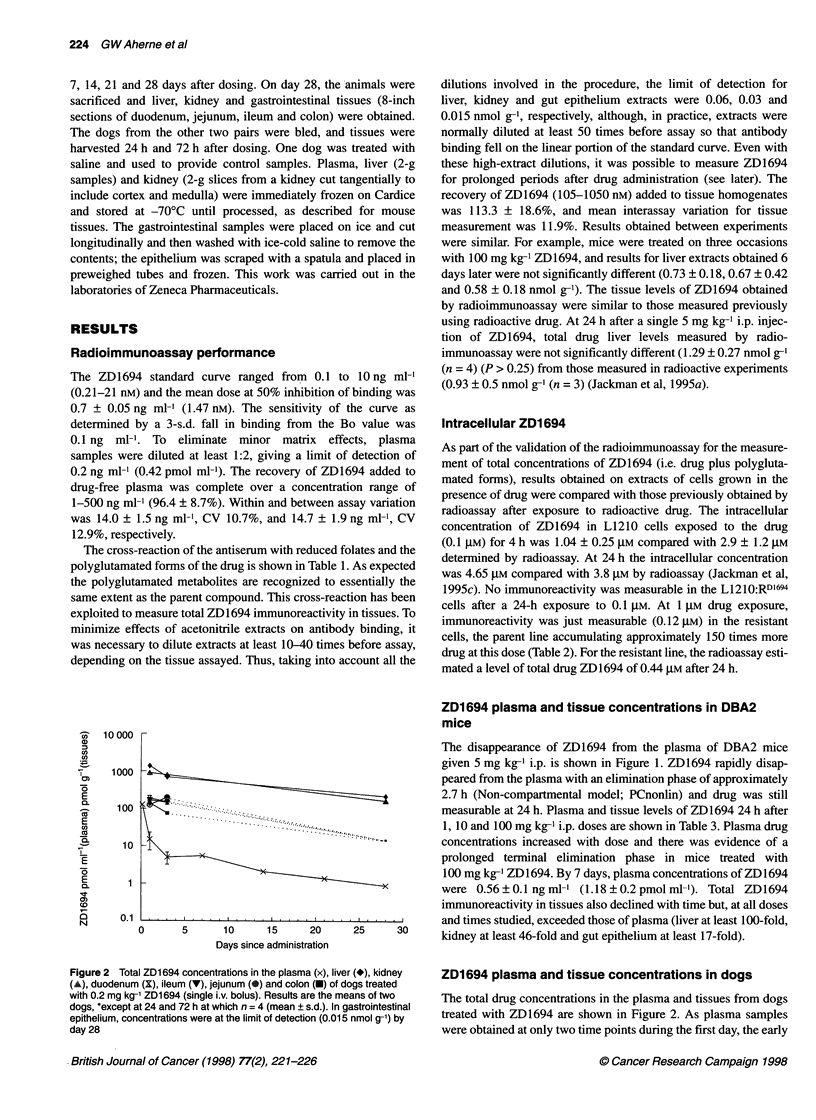

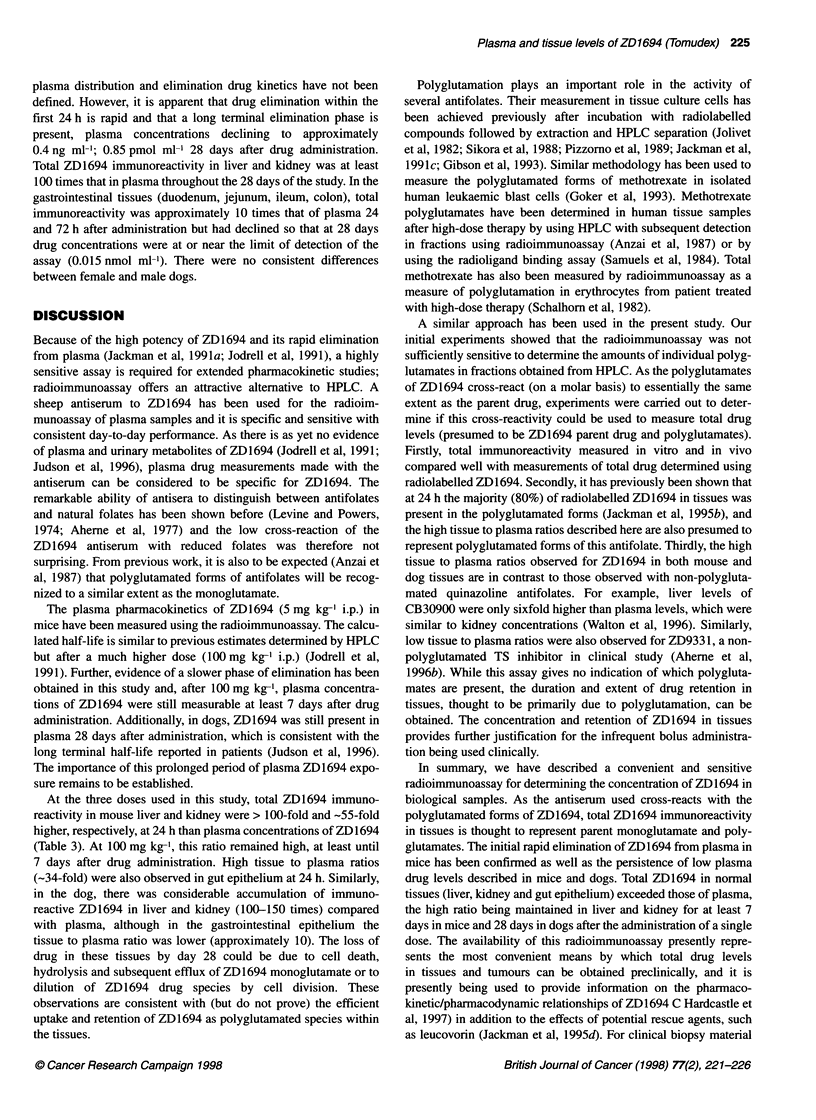

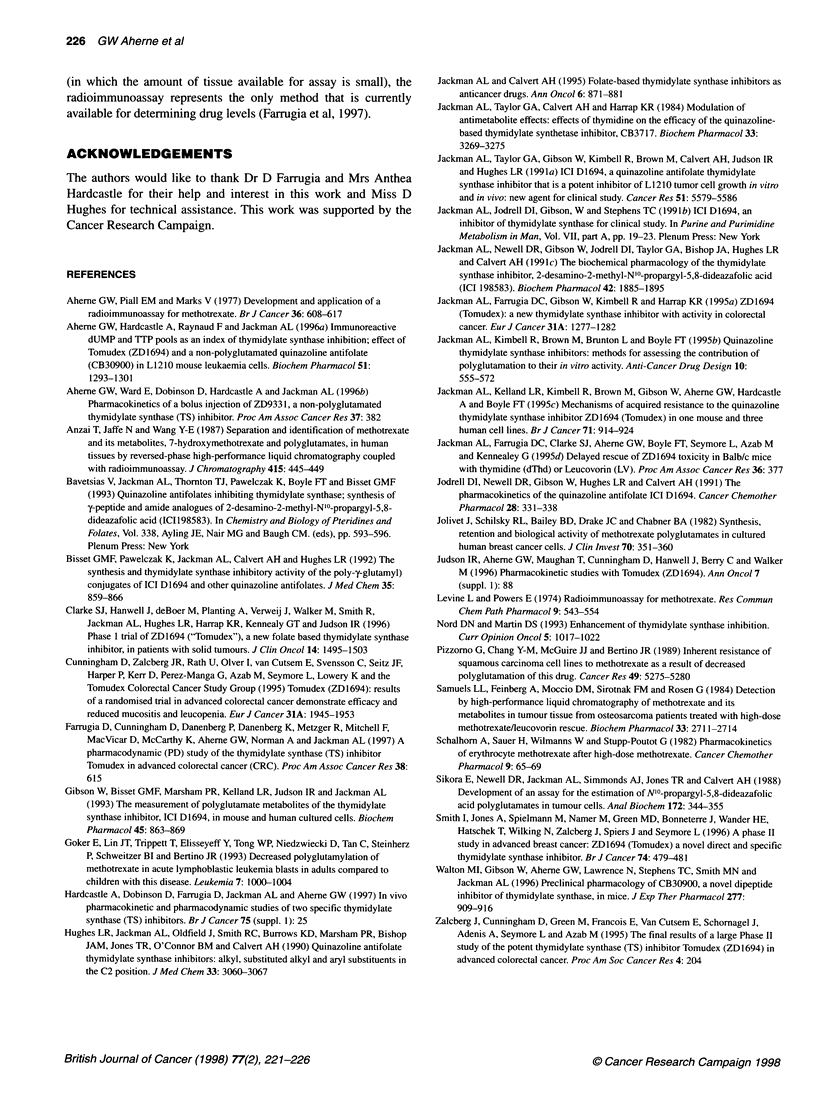


## References

[OCR_00670] Aherne G. W., Hardcastle A., Raynaud F., Jackman A. L. (1996). Immunoreactive dUMP and TTP pools as an index of thymidylate synthase inhibition; effect of tomudex (ZD1694) and a nonpolyglutamated quinazoline antifolate (CB30900) in L1210 mouse leukaemia cells.. Biochem Pharmacol.

[OCR_00666] Aherne G. W., Piall E. M., Marks V. (1977). Development and application of a radioimmunoassay for methotrexate.. Br J Cancer.

[OCR_00682] Anzai T., Jaffe N., Wang Y. M. (1987). Separation and identification of methotrexate and its metabolites, 7-hydroxymethotrexate and polyglutamates, in human tissues by reversed-phase high-performance liquid chromatography coupled with radioimmunoassay.. J Chromatogr.

[OCR_00689] Bavetsias V., Jackman A. L., Thornton T. J., Pawelczak K., Boyle F. T., Bisset G. M. (1993). Quinazoline antifolates inhibiting thymidylate synthase: synthesis of gamma-linked peptide and amide analogues of 2-desamino-2-methyl-N10-propargyl- 5,8-dideazafolic acid (ICI 198583).. Adv Exp Med Biol.

[OCR_00697] Bisset G. M., Pawelczak K., Jackman A. L., Calvert A. H., Hughes L. R. (1992). Syntheses and thymidylate synthase inhibitory activity of the poly-gamma-glutamyl conjugates of N-[5-[N-(3,4-dihydro-2-methyl-4-oxoquinazolin-6-ylmethyl)-N-methylamino ]-2-thenoyl]-L-glutamic acid (ICI D1694) and other quinazoline antifolates.. J Med Chem.

[OCR_00703] Clarke S. J., Hanwell J., de Boer M., Planting A., Verweij J., Walker M., Smith R., Jackman A. L., Hughes L. R., Harrap K. R. (1996). Phase I trial of ZD1694, a new folate-based thymidylate synthase inhibitor, in patients with solid tumors.. J Clin Oncol.

[OCR_00710] Cunningham D., Zalcberg J. R., Rath U., Olver I., Van Cutsem E., Svensson C., Seitz J. F., Harper P., Kerr D., Perez-Manga G. (1995). 'Tomudex' (ZD1694): results of a randomised trial in advanced colorectal cancer demonstrate efficacy and reduced mucositis and leucopenia. The 'Tomudex' Colorectal Cancer Study Group.. Eur J Cancer.

[OCR_00726] Gibson W., Bisset G. M., Marsham P. R., Kelland L. R., Judson I. R., Jackman A. L. (1993). The measurement of polyglutamate metabolites of the thymidylate synthase inhibitor, ICI D1694, in mouse and human cultured cells.. Biochem Pharmacol.

[OCR_00733] Göker E., Lin J. T., Trippett T., Elisseyeff Y., Tong W. P., Niedzwiecki D., Tan C., Steinherz P., Schweitzer B. I., Bertino J. R. (1993). Decreased polyglutamylation of methotrexate in acute lymphoblastic leukemia blasts in adults compared to children with this disease.. Leukemia.

[OCR_00745] Hughes L. R., Jackman A. L., Oldfield J., Smith R. C., Burrows K. D., Marsham P. R., Bishop J. A., Jones T. R., O'Connor B. M., Calvert A. H. (1990). Quinazoline antifolate thymidylate synthase inhibitors: alkyl, substituted alkyl, and aryl substituents in the C2 position.. J Med Chem.

[OCR_00752] Jackman A. L., Calvert A. H. (1995). Folate-based thymidylate synthase inhibitors as anticancer drugs.. Ann Oncol.

[OCR_00792] Jackman A. L., Kelland L. R., Kimbell R., Brown M., Gibson W., Aherne G. W., Hardcastle A., Boyle F. T. (1995). Mechanisms of acquired resistance to the quinazoline thymidylate synthase inhibitor ZD1694 (Tomudex) in one mouse and three human cell lines.. Br J Cancer.

[OCR_00786] Jackman A. L., Kimbell R., Brown M., Brunton L., Boyle F. T. (1995). Quinazoline thymidylate synthase inhibitors: methods for assessing the contribution of polyglutamation to their in vitro activity.. Anticancer Drug Des.

[OCR_00774] Jackman A. L., Newell D. R., Gibson W., Jodrell D. I., Taylor G. A., Bishop J. A., Hughes L. R., Calvert A. H. (1991). The biochemical pharmacology of the thymidylate synthase inhibitor, 2-desamino-2-methyl-N10-propargyl-5,8-dideazafolic acid (ICI 198583).. Biochem Pharmacol.

[OCR_00756] Jackman A. L., Taylor G. A., Calvert A. H., Harrap K. R. (1984). Modulation of anti-metabolite effects. Effects of thymidine on the efficacy of the quinazoline-based thymidylate synthetase inhibitor, CB3717.. Biochem Pharmacol.

[OCR_00762] Jackman A. L., Taylor G. A., Gibson W., Kimbell R., Brown M., Calvert A. H., Judson I. R., Hughes L. R. (1991). ICI D1694, a quinazoline antifolate thymidylate synthase inhibitor that is a potent inhibitor of L1210 tumor cell growth in vitro and in vivo: a new agent for clinical study.. Cancer Res.

[OCR_00803] Jodrell D. I., Newell D. R., Gibson W., Hughes L. R., Calvert A. H. (1991). The pharmacokinetics of the quinazoline antifolate ICI D 1694 in mice and rats.. Cancer Chemother Pharmacol.

[OCR_00808] Jolivet J., Schilsky R. L., Bailey B. D., Drake J. C., Chabner B. A. (1982). Synthesis, retention, and biological activity of methotrexate polyglutamates in cultured human breast cancer cells.. J Clin Invest.

[OCR_00818] Levine L., Powers E. (1974). Radioimmunoassay for methotrexate.. Res Commun Chem Pathol Pharmacol.

[OCR_00822] Nord L. D., Martin D. S. (1993). Enhancement of thymidylate synthase inhibition.. Curr Opin Oncol.

[OCR_00826] Pizzorno G., Chang Y. M., McGuire J. J., Bertino J. R. (1989). Inherent resistance of human squamous carcinoma cell lines to methotrexate as a result of decreased polyglutamylation of this drug.. Cancer Res.

[OCR_00831] Samuels L. L., Feinberg A., Moccio D. M., Sirotnak F. M., Rosen G. (1984). Detection by high-performance liquid chromatography of methotrexate and its metabolites in tumor tissue from osteosarcoma patients treated with high-dose methotrexate/leucovorin rescue.. Biochem Pharmacol.

[OCR_00838] Schalhorn A., Sauer H., Wilmanns W., Stupp-Poutot G. (1982). Pharmacokinetics of erythrocyte methotrexate after high-dose methotrexate.. Cancer Chemother Pharmacol.

[OCR_00843] Sikora E., Newell D. R., Jackman A. L., Simmonds A. J., Jones T. R., Calvert A. H. (1988). Development of an assay for the estimation of N10-propargyl-5,8-dideazafolic acid polyglutamates in tumor cells.. Anal Biochem.

[OCR_00848] Smith I., Jones A., Spielmann M., Namer M., Green M. D., Bonneterre J., Wander H. E., Hatschek T., Wilking N., Zalcberg J. (1996). A phase II study in advanced breast cancer: ZD1694 ('Tomudex') a novel direct and specific thymidylate synthase inhibitor.. Br J Cancer.

[OCR_00855] Walton M. I., Gibson W., Aherne G. W., Lawrence N., Stephens T. C., Smith M. N., Jackman A. L. (1996). Preclinical pharmacology of CB30900, a novel dipeptide inhibitor of thymidylate synthase, in mice.. J Pharmacol Exp Ther.

